# (*E*)-1-{4-[Bis(4-bromo­phen­yl)meth­yl]piperazin-1-yl}-3-(4-eth­oxy­phen­yl)prop-2-en-1-one

**DOI:** 10.1107/S1600536812003819

**Published:** 2012-02-04

**Authors:** Yan Zhong, XiaoPing Zhang, Bin Wu

**Affiliations:** a School of Chemistry and Chemical Engineering, Southeast University, Sipailou No. 2 Nanjing, Nanjing 210096, People’s Republic of China; b Centre of Laboratory Animal, Nanjing Medical University, Hanzhong Road No. 140 Nanjing, Nanjing 210029, People’s Republic of China; c School of Pharmacy, Nanjing Medical University, Hanzhong Road No. 140 Nanjing, Nanjing 210029, People’s Republic of China

## Abstract

In the title compound, C_28_H_28_Br_2_N_2_O_2_, the C=C double bond has an *E* configuration and the piperazine ring has a chair conformation, with the N—C bonds in equatorial orientations. The dihedral angle between the bromo­benzene rings is 83.1 (4)°. In the crystal, mol­ecules are linked by C—H⋯O and C—H⋯Br hydrogen bonds.

## Related literature

For related structures and background to cinnamic acid derivatives, see: Teng *et al.* (2011 ▶); Zhong *et al.* (2012 ▶). For further synthetic details, see: Wu *et al.* (2008 ▶).
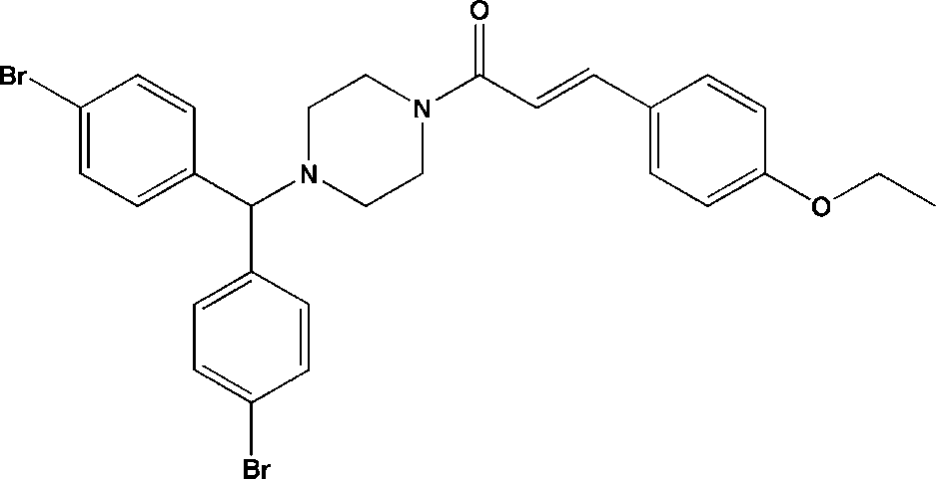


## Experimental

### 

#### Crystal data

C_28_H_28_Br_2_N_2_O_2_
*M* *_r_* = 584.34
Triclinic, 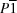

*a* = 10.423 (2) Å
*b* = 10.903 (2) Å
*c* = 12.824 (3) Å
α = 74.29 (3)°
β = 73.56 (3)°
γ = 71.59 (3)°
*V* = 1299.6 (5) Å^3^
*Z* = 2
Mo *K*α radiation
μ = 3.15 mm^−1^
*T* = 293 K
0.20 × 0.10 × 0.10 mm

#### Data collection

Enraf–Nonius CAD-4 diffractometer
Absorption correction: ψ scan (North *et al.*, 1968 ▶) *T*_min_ = 0.572, *T*_max_ = 0.744
5064 measured reflections
4778 independent reflections
2268 reflections with *I* > 2σ(*I*)
*R*_int_ = 0.069
3 standard reflections every 200 reflections intensity decay: 1%

#### Refinement

*R*[*F* ^2^ > 2σ(*F* ^2^)] = 0.068
*wR*(*F* ^2^) = 0.157
*S* = 1.00
4778 reflections
307 parameters
H-atom parameters constrained
Δρ_max_ = 0.57 e Å^−3^
Δρ_min_ = −0.62 e Å^−3^

### 

Data collection: *CAD-4 EXPRESS* (Enraf–Nonius, 1989 ▶); cell refinement: *XCAD4* (Harms & Wocadlo, 1995 ▶); data reduction: *XCAD4*; program(s) used to solve structure: *SHELXS97* (Sheldrick, 2008 ▶); program(s) used to refine structure: *SHELXL97* (Sheldrick, 2008 ▶); molecular graphics: *SHELXTL* (Sheldrick, 2008 ▶); software used to prepare material for publication: *SHELXL97*.

## Supplementary Material

Crystal structure: contains datablock(s) I, global. DOI: 10.1107/S1600536812003819/hb6615sup1.cif

Structure factors: contains datablock(s) I. DOI: 10.1107/S1600536812003819/hb6615Isup2.hkl

Supplementary material file. DOI: 10.1107/S1600536812003819/hb6615Isup3.cml

Additional supplementary materials:  crystallographic information; 3D view; checkCIF report

## Figures and Tables

**Table 1:** Hydrogen-bond geometry (Å, °)

*D*—H⋯*A*	*D*—H	H⋯*A*	*D*⋯*A*	*D*—H⋯*A*
C15—H15*A*⋯Br2^i^	0.97	2.91	3.605 (7)	129
C22—H22*A*⋯O1^ii^	0.93	2.47	3.370 (9)	161

Symmetry codes: (i) 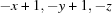; (ii) 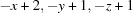.

**Fig. 1.**
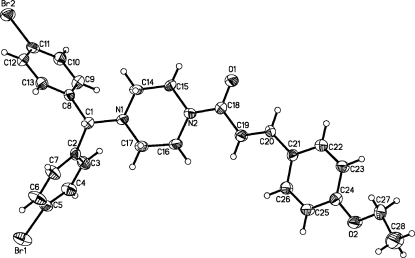
The molecular structure of (I) with displacement ellipsoids for non-H drawn at 70% probability level.

**Fig. 2.**
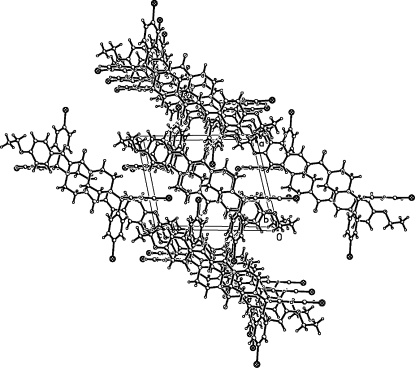
Packing diagram of the title compound.

## supplementary crystallographic information

### Comment

As a continuation of our study of cinnamic acid derivatives (Teng *et al.*, 2011; Zhong *et al.*, 2012), we present here the title compound (I). In (I) (Fig. 1), all bond lengths and angles are normal and correspond to those observed in related compounds (Teng *et al.*, 2011; Zhong *et al.*, 2012). The molecule of (I) exists an E configulation with respect to the C19=C20 ethene bond [1.309 (8)]. The piperazine ring adopts a chair conformation with puchering parameters Q = 0.566 (8), theta = 168.5 (7), phi = 178 (4). In the crystal, molecules are linked by C—H···O and C—H···Br hydrogen bonds.

### Experimental

The synthesis follows the method of Wu *et al.* (2008). The title compound was prepared by stirring a mixture of (*E*)-3-(4-ethoxyphenyl)acrylic acid (0.769 g; 4 mmol), thionyl chloride (2 ml) and dichloromethane (30 ml) for 6 h at room temperature. The solvent was removed under reduced pressure. The residue was dissolved in acetone (15 ml) and reacted with 1-(bis(4-bromophenyl)methyl)iperazine (2.461 g; 6 mmol) in the presence of triethylamine (5 ml) for 12 h at room temperature. The resultant mixture was cooled. The solid, (*E*)-1-(4-(bis(4-bromophenyl)methyl)piperazin-1-yl)-3- (4-ethoxyphenyl)prop-2-en-1-one obtained was filtered and was recrystallized from ethanol. Colourless blocks were grown from an ethanol: acetonitrile:chloroform (1:1:1) solution by slow evaporation at room temperature.

### Refinement

All non-hydrogen atoms were refined anisotropically. All hydrogen atoms were positioned geometrically with C—H distances ranging from 0.93 Å to 0.98 Å and refined as riding on their parent atoms with *U*ĩso~(H) = 1.2 or 1.5*U*~eq~ of the carrier atom.

### Crystal data

**Table:**

C_28_H_28_Br_2_N_2_O_2_	*Z* = 2
*M**_r_* = 584.34	*F*(000) = 592
Triclinic, *P*1	*D*_x_ = 1.493 Mg m^−^^3^
*a* = 10.423 (2) Å	Mo *K*α radiation, λ = 0.71073 Å
*b* = 10.903 (2) Å	Cell parameters from 25 reflections
*c* = 12.824 (3) Å	θ = 9–12°
α = 74.29 (3)°	µ = 3.15 mm^−^^1^
β = 73.56 (3)°	*T* = 293 K
γ = 71.59 (3)°	Block, colorless
*V* = 1299.6 (5) Å^3^	0.20 × 0.10 × 0.10 mm

### Data collection

**Table:**

Enraf–Nonius CAD-4 diffractometer	2268 reflections with *I* > 2σ(*I*)
Radiation source: fine-focus sealed tube	*R*_int_ = 0.069
Graphite monochromator	θ_max_ = 25.4°, θ_min_ = 1.7°
ω/2θ scans	*h* = 0→12
Absorption correction: ψ scan (North *et al.*, 1968)	*k* = −12→13
*T*_min_ = 0.572, *T*_max_ = 0.744	*l* = −14→15
5064 measured reflections	3 standard reflections every 200 reflections
4778 independent reflections	intensity decay: 1%

### Refinement

**Table:**

Refinement on *F*^2^	Primary atom site location: structure-invariant direct methods
Least-squares matrix: full	Secondary atom site location: difference Fourier map
*R*[*F*^2^ > 2σ(*F*^2^)] = 0.068	Hydrogen site location: inferred from neighbouring sites
*wR*(*F*^2^) = 0.157	H-atom parameters constrained
*S* = 1.00	*w* = 1/[σ^2^(*F*_o_^2^) + (0.063*P*)^2^] where *P* = (*F*_o_^2^ + 2*F*_c_^2^)/3
4778 reflections	(Δ/σ)_max_ < 0.001
307 parameters	Δρ_max_ = 0.57 e Å^−^^3^
0 restraints	Δρ_min_ = −0.62 e Å^−^^3^

### Special details

**Table:**

Geometry. All e.s.d.'s (except the e.s.d. in the dihedral angle between two l.s. planes) are estimated using the full covariance matrix. The cell e.s.d.'s are taken into account individually in the estimation of e.s.d.'s in distances, angles and torsion angles; correlations between e.s.d.'s in cell parameters are only used when they are defined by crystal symmetry. An approximate (isotropic) treatment of cell e.s.d.'s is used for estimating e.s.d.'s involving l.s. planes.
Refinement. Refinement of *F*^2^ against ALL reflections. The weighted *R*-factor *wR* and goodness of fit *S* are based on *F*^2^, conventional *R*-factors *R* are based on *F*, with *F* set to zero for negative *F*^2^. The threshold expression of *F*^2^ > σ(*F*^2^) is used only for calculating *R*-factors(gt) *etc*. and is not relevant to the choice of reflections for refinement. *R*-factors based on *F*^2^ are statistically about twice as large as those based on *F*, and *R*- factors based on ALL data will be even larger.

### Fractional atomic coordinates and isotropic or equivalent isotropic displacement parameters (Å^2^)

**Table:**

	*x*	*y*	*z*	*U*_iso_*/*U*_eq_	
Br1	−0.31209 (9)	0.97529 (9)	0.41018 (8)	0.0772 (4)	
N1	0.4018 (6)	0.7583 (6)	0.2995 (4)	0.0444 (15)	
O1	0.8264 (5)	0.5375 (5)	0.4350 (4)	0.0636 (15)	
C1	0.3172 (7)	0.8540 (7)	0.2261 (5)	0.0409 (17)	
H1A	0.3478	0.9354	0.2026	0.049*	
Br2	0.33465 (8)	0.64618 (9)	−0.18076 (7)	0.0655 (3)	
O2	0.8295 (5)	0.7395 (5)	1.0157 (4)	0.0644 (16)	
N2	0.6065 (6)	0.6446 (5)	0.4291 (4)	0.0471 (15)	
C2	0.1658 (7)	0.8848 (7)	0.2841 (5)	0.0395 (17)	
C3	0.1050 (7)	0.7871 (7)	0.3459 (6)	0.0499 (19)	
H3A	0.1594	0.7011	0.3601	0.060*	
C4	−0.0386 (8)	0.8139 (8)	0.3888 (6)	0.058 (2)	
H4A	−0.0797	0.7472	0.4321	0.069*	
C5	−0.1149 (7)	0.9390 (8)	0.3653 (6)	0.052 (2)	
C6	−0.0569 (8)	1.0417 (8)	0.3059 (7)	0.070 (3)	
H6A	−0.1109	1.1281	0.2927	0.084*	
C7	0.0832 (7)	1.0110 (7)	0.2674 (6)	0.060 (2)	
H7A	0.1246	1.0789	0.2280	0.072*	
C8	0.3295 (6)	0.8018 (7)	0.1241 (6)	0.0378 (17)	
C9	0.3366 (7)	0.6702 (7)	0.1330 (6)	0.0488 (19)	
H9A	0.3403	0.6124	0.2010	0.059*	
C10	0.3382 (7)	0.6244 (7)	0.0428 (6)	0.0464 (18)	
H10A	0.3439	0.5359	0.0492	0.056*	
C11	0.3315 (7)	0.7093 (8)	−0.0554 (6)	0.0463 (19)	
C12	0.3232 (7)	0.8403 (8)	−0.0682 (6)	0.052 (2)	
H12A	0.3177	0.8977	−0.1362	0.063*	
C13	0.3234 (7)	0.8846 (7)	0.0228 (6)	0.0473 (19)	
H13A	0.3193	0.9729	0.0153	0.057*	
C14	0.5494 (7)	0.7223 (7)	0.2480 (6)	0.051 (2)	
H14A	0.5864	0.7983	0.2320	0.062*	
H14B	0.5613	0.6967	0.1784	0.062*	
C15	0.6267 (7)	0.6126 (7)	0.3213 (5)	0.050 (2)	
H15A	0.5958	0.5342	0.3315	0.060*	
H15B	0.7246	0.5936	0.2867	0.060*	
C16	0.4626 (7)	0.6980 (8)	0.4790 (6)	0.053 (2)	
H16A	0.4580	0.7315	0.5431	0.064*	
H16B	0.4153	0.6281	0.5043	0.064*	
C17	0.3915 (7)	0.8055 (7)	0.3996 (6)	0.051 (2)	
H17A	0.2948	0.8364	0.4344	0.061*	
H17B	0.4334	0.8788	0.3791	0.061*	
C18	0.7144 (8)	0.5993 (7)	0.4799 (6)	0.0458 (18)	
C19	0.6976 (7)	0.6322 (7)	0.5881 (6)	0.0487 (19)	
H19A	0.6105	0.6753	0.6236	0.058*	
C20	0.8030 (7)	0.6019 (7)	0.6349 (5)	0.0441 (18)	
H20A	0.8859	0.5529	0.5984	0.053*	
C21	0.8080 (7)	0.6342 (7)	0.7358 (5)	0.0402 (17)	
C22	0.9325 (7)	0.6010 (7)	0.7672 (6)	0.0506 (19)	
H22A	1.0115	0.5553	0.7240	0.061*	
C23	0.9439 (7)	0.6326 (7)	0.8587 (6)	0.051 (2)	
H23A	1.0299	0.6093	0.8770	0.062*	
C24	0.8290 (7)	0.6989 (7)	0.9245 (6)	0.0450 (18)	
C25	0.7031 (7)	0.7326 (7)	0.8959 (6)	0.049 (2)	
H25A	0.6238	0.7761	0.9403	0.059*	
C26	0.6947 (7)	0.7022 (7)	0.8028 (6)	0.0491 (19)	
H26A	0.6092	0.7283	0.7833	0.059*	
C27	0.9597 (8)	0.7367 (8)	1.0333 (6)	0.063 (2)	
H27A	1.0113	0.7814	0.9664	0.075*	
H27B	1.0134	0.6460	1.0502	0.075*	
C28	0.9381 (10)	0.8017 (10)	1.1251 (8)	0.106 (4)	
H28A	1.0262	0.7982	1.1370	0.159*	
H28B	0.8869	0.7574	1.1912	0.159*	
H28C	0.8871	0.8921	1.1073	0.159*	

### Atomic displacement parameters (Å^2^)

**Table:**

	*U*^11^	*U*^22^	*U*^33^	*U*^12^	*U*^13^	*U*^23^
Br1	0.0486 (5)	0.0800 (7)	0.0987 (8)	−0.0114 (5)	0.0050 (5)	−0.0390 (6)
N1	0.044 (4)	0.055 (4)	0.036 (3)	−0.006 (3)	−0.008 (3)	−0.021 (3)
O1	0.050 (3)	0.087 (4)	0.057 (3)	0.001 (3)	−0.016 (3)	−0.036 (3)
C1	0.045 (4)	0.043 (5)	0.036 (4)	−0.014 (4)	−0.008 (4)	−0.008 (3)
Br2	0.0573 (6)	0.0891 (7)	0.0612 (6)	−0.0102 (5)	−0.0137 (4)	−0.0422 (5)
O2	0.045 (3)	0.101 (4)	0.062 (3)	−0.014 (3)	−0.012 (3)	−0.046 (3)
N2	0.043 (4)	0.052 (4)	0.045 (4)	0.002 (3)	−0.010 (3)	−0.023 (3)
C2	0.042 (4)	0.035 (4)	0.038 (4)	−0.007 (4)	−0.014 (3)	0.000 (3)
C3	0.047 (5)	0.033 (4)	0.064 (5)	−0.004 (4)	−0.012 (4)	−0.008 (4)
C4	0.054 (5)	0.051 (5)	0.063 (5)	−0.012 (4)	−0.005 (4)	−0.015 (4)
C5	0.040 (4)	0.057 (5)	0.064 (5)	0.001 (4)	−0.013 (4)	−0.033 (4)
C6	0.050 (5)	0.043 (5)	0.103 (7)	−0.003 (4)	0.002 (5)	−0.024 (5)
C7	0.043 (5)	0.043 (5)	0.082 (6)	−0.008 (4)	−0.005 (4)	−0.005 (4)
C8	0.032 (4)	0.042 (5)	0.040 (4)	−0.007 (3)	−0.011 (3)	−0.009 (4)
C9	0.058 (5)	0.053 (5)	0.040 (4)	−0.020 (4)	−0.014 (4)	−0.007 (4)
C10	0.055 (5)	0.036 (4)	0.049 (5)	−0.010 (4)	−0.014 (4)	−0.009 (4)
C11	0.032 (4)	0.056 (5)	0.056 (5)	−0.003 (4)	−0.008 (4)	−0.030 (4)
C12	0.052 (5)	0.067 (6)	0.035 (4)	−0.008 (4)	−0.013 (4)	−0.013 (4)
C13	0.054 (5)	0.036 (4)	0.047 (5)	−0.005 (4)	−0.013 (4)	−0.006 (4)
C14	0.042 (4)	0.064 (5)	0.048 (5)	−0.003 (4)	−0.003 (4)	−0.030 (4)
C15	0.058 (5)	0.057 (5)	0.037 (4)	−0.001 (4)	−0.017 (4)	−0.023 (4)
C16	0.044 (5)	0.074 (6)	0.043 (5)	−0.007 (4)	−0.004 (4)	−0.029 (4)
C17	0.039 (4)	0.067 (6)	0.050 (5)	−0.005 (4)	−0.007 (4)	−0.030 (4)
C18	0.047 (5)	0.051 (5)	0.040 (4)	−0.009 (4)	−0.012 (4)	−0.013 (4)
C19	0.041 (4)	0.060 (5)	0.050 (5)	−0.009 (4)	−0.011 (4)	−0.023 (4)
C20	0.041 (4)	0.045 (5)	0.044 (4)	0.001 (4)	−0.017 (4)	−0.012 (4)
C21	0.034 (4)	0.052 (5)	0.037 (4)	−0.013 (4)	−0.009 (3)	−0.010 (3)
C22	0.046 (5)	0.056 (5)	0.048 (5)	−0.006 (4)	−0.009 (4)	−0.018 (4)
C23	0.037 (4)	0.068 (6)	0.057 (5)	−0.008 (4)	−0.011 (4)	−0.031 (4)
C24	0.045 (5)	0.052 (5)	0.046 (4)	−0.021 (4)	−0.010 (4)	−0.011 (4)
C25	0.036 (4)	0.067 (6)	0.055 (5)	−0.016 (4)	−0.005 (4)	−0.030 (4)
C26	0.039 (4)	0.062 (5)	0.055 (5)	−0.014 (4)	−0.013 (4)	−0.023 (4)
C27	0.057 (5)	0.078 (6)	0.063 (6)	−0.013 (5)	−0.029 (4)	−0.017 (5)
C28	0.093 (7)	0.147 (10)	0.113 (8)	−0.015 (7)	−0.041 (6)	−0.081 (8)

### Geometric parameters (Å, º)

**Table:**

Br1—C5	1.911 (7)	C13—H13A	0.9300
N1—C1	1.452 (8)	C14—C15	1.475 (9)
N1—C14	1.463 (8)	C14—H14A	0.9700
N1—C17	1.473 (8)	C14—H14B	0.9700
O1—C18	1.217 (8)	C15—H15A	0.9700
C1—C2	1.514 (9)	C15—H15B	0.9700
C1—C8	1.520 (9)	C16—C17	1.480 (9)
C1—H1A	0.9800	C16—H16A	0.9700
Br2—C11	1.901 (7)	C16—H16B	0.9700
O2—C24	1.360 (7)	C17—H17A	0.9700
O2—C27	1.428 (8)	C17—H17B	0.9700
N2—C18	1.350 (8)	C18—C19	1.476 (9)
N2—C16	1.451 (8)	C19—C20	1.309 (8)
N2—C15	1.458 (7)	C19—H19A	0.9300
C2—C3	1.360 (9)	C20—C21	1.448 (9)
C2—C7	1.370 (9)	C20—H20A	0.9300
C3—C4	1.404 (9)	C21—C22	1.378 (9)
C3—H3A	0.9300	C21—C26	1.379 (9)
C4—C5	1.346 (10)	C22—C23	1.355 (9)
C4—H4A	0.9300	C22—H22A	0.9300
C5—C6	1.380 (10)	C23—C24	1.371 (9)
C6—C7	1.364 (9)	C23—H23A	0.9300
C6—H6A	0.9300	C24—C25	1.375 (9)
C7—H7A	0.9300	C25—C26	1.354 (8)
C8—C13	1.372 (9)	C25—H25A	0.9300
C8—C9	1.388 (9)	C26—H26A	0.9300
C9—C10	1.373 (9)	C27—C28	1.462 (10)
C9—H9A	0.9300	C27—H27A	0.9700
C10—C11	1.351 (9)	C27—H27B	0.9700
C10—H10A	0.9300	C28—H28A	0.9600
C11—C12	1.371 (10)	C28—H28B	0.9600
C12—C13	1.379 (9)	C28—H28C	0.9600
C12—H12A	0.9300		
			
C1—N1—C14	113.9 (5)	N2—C15—H15A	109.4
C1—N1—C17	112.4 (5)	C14—C15—H15A	109.4
C14—N1—C17	106.2 (5)	N2—C15—H15B	109.4
N1—C1—C2	111.0 (5)	C14—C15—H15B	109.4
N1—C1—C8	111.0 (5)	H15A—C15—H15B	108.0
C2—C1—C8	107.5 (5)	N2—C16—C17	111.6 (6)
N1—C1—H1A	109.1	N2—C16—H16A	109.3
C2—C1—H1A	109.1	C17—C16—H16A	109.3
C8—C1—H1A	109.1	N2—C16—H16B	109.3
C24—O2—C27	118.1 (5)	C17—C16—H16B	109.3
C18—N2—C16	126.7 (6)	H16A—C16—H16B	108.0
C18—N2—C15	118.0 (5)	N1—C17—C16	110.3 (6)
C16—N2—C15	113.8 (5)	N1—C17—H17A	109.6
C3—C2—C7	118.0 (7)	C16—C17—H17A	109.6
C3—C2—C1	121.2 (6)	N1—C17—H17B	109.6
C7—C2—C1	120.6 (6)	C16—C17—H17B	109.6
C2—C3—C4	121.2 (7)	H17A—C17—H17B	108.1
C2—C3—H3A	119.4	O1—C18—N2	120.7 (6)
C4—C3—H3A	119.4	O1—C18—C19	120.0 (7)
C5—C4—C3	118.0 (7)	N2—C18—C19	119.2 (6)
C5—C4—H4A	121.0	C20—C19—C18	121.2 (7)
C3—C4—H4A	121.0	C20—C19—H19A	119.4
C4—C5—C6	122.6 (7)	C18—C19—H19A	119.4
C4—C5—Br1	118.9 (6)	C19—C20—C21	128.8 (7)
C6—C5—Br1	118.4 (6)	C19—C20—H20A	115.6
C7—C6—C5	117.1 (7)	C21—C20—H20A	115.6
C7—C6—H6A	121.5	C22—C21—C26	116.2 (6)
C5—C6—H6A	121.5	C22—C21—C20	119.8 (6)
C6—C7—C2	123.0 (7)	C26—C21—C20	123.9 (6)
C6—C7—H7A	118.5	C23—C22—C21	122.3 (7)
C2—C7—H7A	118.5	C23—C22—H22A	118.8
C13—C8—C9	118.0 (6)	C21—C22—H22A	118.8
C13—C8—C1	121.3 (6)	C22—C23—C24	120.1 (7)
C9—C8—C1	120.5 (6)	C22—C23—H23A	119.9
C10—C9—C8	120.9 (7)	C24—C23—H23A	119.9
C10—C9—H9A	119.6	O2—C24—C23	125.0 (6)
C8—C9—H9A	119.6	O2—C24—C25	116.0 (6)
C11—C10—C9	119.3 (7)	C23—C24—C25	119.0 (6)
C11—C10—H10A	120.4	C26—C25—C24	119.9 (7)
C9—C10—H10A	120.4	C26—C25—H25A	120.1
C10—C11—C12	122.1 (7)	C24—C25—H25A	120.1
C10—C11—Br2	119.4 (6)	C25—C26—C21	122.5 (7)
C12—C11—Br2	118.5 (6)	C25—C26—H26A	118.7
C11—C12—C13	118.0 (7)	C21—C26—H26A	118.7
C11—C12—H12A	121.0	O2—C27—C28	110.1 (7)
C13—C12—H12A	121.0	O2—C27—H27A	109.7
C8—C13—C12	121.8 (7)	C28—C27—H27A	109.7
C8—C13—H13A	119.1	O2—C27—H27B	109.6
C12—C13—H13A	119.1	C28—C27—H27B	109.7
N1—C14—C15	111.2 (6)	H27A—C27—H27B	108.2
N1—C14—H14A	109.4	C27—C28—H28A	109.5
C15—C14—H14A	109.4	C27—C28—H28B	109.5
N1—C14—H14B	109.4	H28A—C28—H28B	109.5
C15—C14—H14B	109.4	C27—C28—H28C	109.5
H14A—C14—H14B	108.0	H28A—C28—H28C	109.5
N2—C15—C14	111.3 (5)	H28B—C28—H28C	109.5
			
C14—N1—C1—C2	179.1 (5)	C1—N1—C14—C15	−172.4 (5)
C17—N1—C1—C2	−60.1 (7)	C17—N1—C14—C15	63.3 (7)
C14—N1—C1—C8	59.5 (7)	C18—N2—C15—C14	−144.3 (7)
C17—N1—C1—C8	−179.7 (5)	C16—N2—C15—C14	48.3 (8)
N1—C1—C2—C3	−45.3 (8)	N1—C14—C15—N2	−56.4 (8)
C8—C1—C2—C3	76.4 (8)	C18—N2—C16—C17	145.1 (7)
N1—C1—C2—C7	140.7 (7)	C15—N2—C16—C17	−48.8 (8)
C8—C1—C2—C7	−97.7 (8)	C1—N1—C17—C16	171.7 (6)
C7—C2—C3—C4	1.8 (11)	C14—N1—C17—C16	−63.1 (7)
C1—C2—C3—C4	−172.4 (6)	N2—C16—C17—N1	56.8 (8)
C2—C3—C4—C5	1.1 (11)	C16—N2—C18—O1	168.1 (7)
C3—C4—C5—C6	−3.2 (11)	C15—N2—C18—O1	2.5 (11)
C3—C4—C5—Br1	174.0 (5)	C16—N2—C18—C19	−15.5 (11)
C4—C5—C6—C7	2.3 (12)	C15—N2—C18—C19	178.9 (6)
Br1—C5—C6—C7	−174.9 (6)	O1—C18—C19—C20	3.4 (11)
C5—C6—C7—C2	0.9 (12)	N2—C18—C19—C20	−173.0 (7)
C3—C2—C7—C6	−2.8 (12)	C18—C19—C20—C21	175.2 (7)
C1—C2—C7—C6	171.4 (7)	C19—C20—C21—C22	−175.2 (8)
N1—C1—C8—C13	−146.2 (6)	C19—C20—C21—C26	2.2 (12)
C2—C1—C8—C13	92.2 (7)	C26—C21—C22—C23	0.2 (11)
N1—C1—C8—C9	38.8 (8)	C20—C21—C22—C23	177.8 (7)
C2—C1—C8—C9	−82.8 (8)	C21—C22—C23—C24	0.6 (12)
C13—C8—C9—C10	0.4 (10)	C27—O2—C24—C23	12.7 (11)
C1—C8—C9—C10	175.5 (6)	C27—O2—C24—C25	−164.7 (7)
C8—C9—C10—C11	−0.6 (11)	C22—C23—C24—O2	−177.5 (7)
C9—C10—C11—C12	0.1 (11)	C22—C23—C24—C25	−0.1 (11)
C9—C10—C11—Br2	179.7 (5)	O2—C24—C25—C26	176.4 (6)
C10—C11—C12—C13	0.7 (11)	C23—C24—C25—C26	−1.2 (11)
Br2—C11—C12—C13	−178.9 (5)	C24—C25—C26—C21	2.1 (12)
C9—C8—C13—C12	0.4 (10)	C22—C21—C26—C25	−1.6 (11)
C1—C8—C13—C12	−174.7 (6)	C20—C21—C26—C25	−179.1 (7)
C11—C12—C13—C8	−1.0 (10)	C24—O2—C27—C28	171.6 (7)

### Hydrogen-bond geometry (Å, º)

**Table:**

*D*—H···*A*	*D*—H	H···*A*	*D*···*A*	*D*—H···*A*
C15—H15*A*···Br2^i^	0.97	2.91	3.605 (7)	129
C22—H22*A*···O1^ii^	0.93	2.47	3.370 (9)	161

Symmetry codes: (i) −*x*+1, −*y*+1, −*z*; (ii) −*x*+2, −*y*+1, −*z*+1.
